# Decoding HPV: disease stage and treatment insights

**DOI:** 10.1002/1878-0261.13661

**Published:** 2024-05-07

**Authors:** Yeliz Yılmaz

**Affiliations:** ^1^ Izmir Biomedicine and Genome Center Turkey

## Abstract

An outlook of human papilloma virus (HPV) in oncogenesis and its role in therapy. The integrated HPV DNA can induce transcriptional upregulation in vicinity or transcription of oncogenes through long‐range chromatin interactions [Singh AK et al. (2023) *Mol Oncol*, https://doi.org/10.1002/1878-0261.13559] and the level of circulating cell‐free HPV DNA is related to disease severity [Bønløkke S et al. (2022) *Cells*, https://doi.org/10.3390/cells11142170]. In addition, plasma IL‐8/IL‐18 levels can be used as biomarkers for predicting therapy response in HPV‐related cancers [Bønløkke S et al. (2023) *Mol Oncol*, https://doi.org/10.1002/1878-0261.13538].

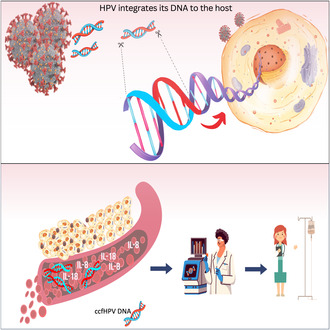

**Abbreviations**
ccf: circulating cell‐free
HPV: human papilloma virus
IL: interleukin
NGS: next‐generation sequencing
PBMCs: peripheral blood mononuclear cells
TADs: topologically associating domains

Papilloma viruses are found vastly in the environment and can infect animal skin and mucosa. Human papilloma virus (HPV), which has more than 200 types, can be the major cause of several cancer types [1]. Although many types of HPV infections are controlled and cleared by the innate immune system within 2 years, persistent HPV infections are known to induce carcinogenesis [2, 3]. Among these persistent infectors, HPV16 is the major player and found in 50–70% of the cases followed by HPV18 found in 7–20% of the cases [3]. Three recent studies published in *Molecular Oncology* focused on the effects of HPV integration, patient stratification, and response to therapy (Fig. 1).

**Fig. 1:**
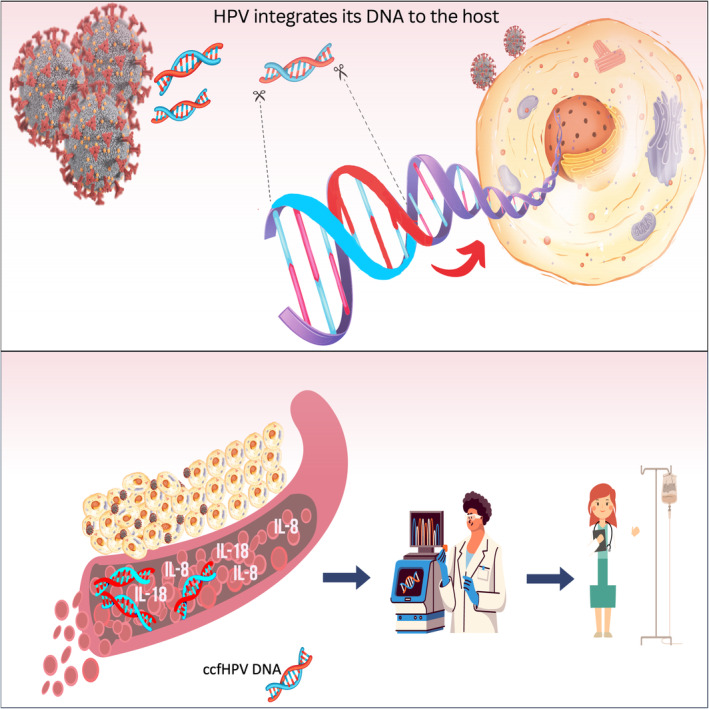
An outlook of HPV in oncogenesis and its role in therapy. The integrated HPV DNA can induce transcriptional upregulation in vicinity or transcription of oncogenes through long‐range chromatin interactions [4] and the level of ccfHPV DNA is related to disease severity [7]. In addition, plasma IL‐8/IL‐18 levels can be used as biomarkers for predicting therapy response in HPV‐related cancers [8].

In the current issue of *Molecular Oncology*, Singh et al. [4] focused on HPV integration into the host genome and investigated cis‐regulatory effect on chromatin and gene regulation. Using genome‐wide DNA‐based approaches, the authors analysed HPV integration locations in the host genome of patients with cervical cancer. They further identified that HPV integrations are enriched in transcriptionally active chromatin regions in the tumour genome and in turn upregulate transcriptional activity in integration vicinity.

Next, Singh et al. focused on topologically associating domains (TADs), which are distinct regulatory chromatin segments that enable interaction within the region while insulating from the interactions across its boundaries [5]. Singh et al. demonstrated that only TADs with HPV integrations are more related to enhanced gene expression and certain HPV subtypes integrate recurrently to specific TADs. Importantly, some recurrently HPV‐integrated TADs were associated with the preferential enhancement of the oncogenes, including Myc, PVT1, ERBB2 and TP63.

Further analysis showed that the effects of HPV integration on gene expression were not limited to close proximity of the integration site but also extended to longer distances due to chromatin looping. Indeed, a potentially strong chromatin interaction between MYC/PVT1 promoters and HPV18 DNA integration (nearly 500 kb apart) was identified. Moreover, the analysis showed that HPV integration‐mediated chromatin interactions are not limited to the same TAD and are in addition haplotype‐specific (HPV18 integration was found in only one of the two haplotypes of chromosome 8). This haplotype‐specific cis‐regulatory activity mediated by HPV integration leads to allele‐specific overexpression of super enhancers and oncogenes within the same TAD. Taken together, the findings of Singh et al. highlight that HPV integrations display cis‐regulatory activity by altering the host's chromatin structure and enable the overexpression of oncogenes that drive carcinogenesis.

Previously it was also shown that non‐integrated HPV DNA (especially for HPV16) might be found in cervical cancer in a pure episomal form [6]. Recently, circulating cell‐free HPV (ccfHPV) DNA was identified in the plasma from patients with cervical cancer and its levels were related to tumour burden in advanced stage cervical cancer [7]. In this issue of *Molecular Oncology*, Bønløkke et al. [8] assessed the integration status of HPV16 and investigated whether plasma ccfHPV DNA could be a marker for disease severity using next‐generation sequencing (NGS) HPV genotyping panels.

Bønløkke et al. first performed HPV genotyping analysis of cervical tissue from 139 patients with cervical cancer using INNO‐LiPA assay and the generated NGS HPV genotyping panel and compared the results. They found that results from both assays were comparable. Therefore, continuing with the NGS HPV genotyping panel, the status of ccfHPV DNA was successfully investigated in pre‐treatment plasma from 139 patients with cervical cancer (including primary surgery, primary surgary + adjuvant oncological therapy and primary oncological therapy groups). The integration status of HPV16 was further examined in tissues from 67 patients with HPV16‐related disease in the NGS HPV16 panel. Further analysis among primary oncology patients led the researchers to find higher levels of HPV16 integration in ccfHPV‐negative patients compared to ccfHPV‐positive patients. Disease severity and ccfHPV16 levels were further studied in a primary cohort of patients with HPV integration. Bønløkke et al. found that the presence of ccfHPV was significantly related to higher disease stages, whereas correlation with tumour size was not evident. This study highlights the use of NGS HPV genotyping for detecting ccfHPV DNA in patients with cervical cancer, and its relation with both disease severity and patient stratification for primary oncological therapy.

Furthermore, in the current issue of *Molecular Oncology*, Bozorgmehr et al. [9] focused on the response of HPV‐infected patients with various types of cancer to a treatment course combining epigenetic inhibitor (valproic acid) and immunotherapy (avelumab). They investigated the transcriptomic profile of patients' peripheral blood cells who received valproic acid (histone deacetylase inhibitor) and avelumab (anti‐PD‐L1), as a part of the LATENT Clinical Trial study. Bulk RNA sequencing of peripheral blood mononuclear cells (PBMCs) enabled the researchers to profile the transcriptome of responsive and non‐responsive patients. The PBMCs were collected from patients and sequenced at four time points throughout the study facilitating a longitudinal peripheral blood transcriptome follow‐up.

Enriched signalling pathway analysis highlighted the downregulation of interleukin‐8 (IL‐8), interleukin‐18 (IL‐18) and vascular endothelial growth factor receptor pathways, and the upregulation of tumour necrosis factor and haptoglobin pathways in the responsive group at the beginning of the treatment. Importantly, in non‐responders, valproic acid treatment downregulated genes of the JAK/STAT pathway and associated cytokines, in addition to genes of the glycolysis and gluconeogenesis pathways during the initial phase of treatment.

When comparing the treatment endpoint to baseline, IL‐8 signalling and neutrophil extracellular trap formation pathways were found to be enriched in non‐responsive patients, whereas IL‐8 signalling was significantly inhibited in the responsive group. These findings suggest that poor clinical response to avelumab might be related to the upregulation of IL‐8 signalling and myeloid cell functions. When immune cell fractions were analysed, myeloid cells were expanded in non‐responders, whereas CD8^+^ T cells were increased in responders at endpoint. Finally, the researchers evaluated the cytokine and chemokine profiles of patients by enzyme‐linked immunosorbent assay and multiplex assay and reported that IL‐8 and IL‐18 were significantly higher in non‐responders. In conclusion, Bozorgmehr et al. highlighted the potential role of IL‐8 and IL‐18 concentrations, and circulating myeloid cells in predicting response to therapy in HPV‐infected carcinoma patients.

Studies like those of Singh et al., Bønløkke et al. and Bozorgmehr et al. would enable researchers not only to understand the mechanisms behind HPV in carcinogenesis, but also to develop more precise treatment and screening strategies for patients with cancer.

## Conflict of interest

The author declares no conflict of interest.
